# Phenolic and Aroma Changes of Red and White Wines during Aging Induced by High Hydrostatic Pressure

**DOI:** 10.3390/foods9081034

**Published:** 2020-08-01

**Authors:** Katarina Lukić, Natka Ćurko, Marina Tomašević, Karin Kovačević Ganić

**Affiliations:** Faculty of Food Technology and Biotechnology, University of Zagreb, Pierottijeva 6, 10000 Zagreb, Croatia; katarina.lukic@pbf.unizg.hr (K.L.); natka.curko@pbf.unizg.hr (N.Ć.); marina.tomasevic@pbf.unizg.hr (M.T.)

**Keywords:** high hydrostatic pressure, wine, phenolics, aroma, aging, SO_2_ content, glutathione

## Abstract

The aim of this study was to investigate use of high hydrostatic pressure (HHP) along with different antioxidants (glutathione and SO_2_) as an alternative method for wine preservation and production of low-SO_2_ wines. In the first phase of the study, low-SO_2,_ young red and white wines were pressurized at three pressure levels (200, 400 and 600 MPa) for 5, 15 and 25 min at room temperature, and analyzed immediately after treatments. Additionally, for the wine aging experiment, red and white wines with standard-SO_2_, low-SO_2_+glutathione and low-SO_2_ content were treated with HHP treatment (200 MPa/5 min) and stored for 12 months in bottles. Color parameters, phenolic and aroma compounds were determined. The sensory evaluation was also conducted. HHP showed very slight, but statistically significant changes in the chemical composition of both red and white wine right after the treatment, and the main variations observed were related to the different pressures applied. Furthermore, during aging, most of the differences observed in chemical composition of pressurized wines, both red and white, were statistically significant, and greater in wines with a lower content of antioxidants. However, after 12 months of aging, some differences between unpressurized and pressurized samples with standard SO_2_ content were lost, primarily in aroma compounds for red wine and in color and phenolics for white wine. Additionally, similar values were obtained for mentioned characteristics of red and white wines in pressurized samples with standard SO_2_ and low SO_2_+glutathione, indicating that HHP in combination with glutathione and lower doses of SO_2_ might potentially preserve wine. The sensory analysis confirmed less pronounced changes in the sensory attributes of pressurized wines with higher concentration of antioxidants. Furthermore, the treatments applied had a slightly higher effect on the sensory properties of white wine.

## 1. Introduction

High hydrostatic pressure (HHP) is one of the most researched, nonthermal techniques for preserving and modifying food products in the last decade. In general, the HHP treatment itself involves the subjection of food, with or without packaging, to high pressure in the range of 100 to 600 MPa [1]. This technique characterizes a minimal increase of temperature, as well as a small effect on low molecular weight compounds during processing [2]. The primary goal of its use is to achieve inactivation of undesirable microorganisms and enzymes with minimal effect on the sensory and nutritional characteristics of the treated product. Therefore, research related to the application of HHP in winemaking have mainly been focused on the microbial control of wine [3,4,5,6,7]. However, in order to achieve full HHP potential for wine industry application, the effect of HHP on the overall wine quality must not be disregarded. Previous studies have shown that HHP does not markedly affect the basic physicochemical properties of wine immediately after processing [3,8,9]. On the other hand, Buzrul [4] and Tao et al. [10] reported that HHP processing at extreme conditions (650 MPa for 1 and 2 h) resulted in changes of physicochemical and sensory properties of wines. Additionally, some investigations revealed that HHP influenced the long-term physicochemical and sensory properties of wines through promotion of reactions associated with those observed during wine aging [9,11,12,13]. According to Santos et al. [9], HHP seems to be a more adequate processing technique for red wines than white wines, since its effect on color properties was only positive for red wines. It was found that HHP accelerates the wine aging process, since it promotes various chemical reactions, namely condensation and oxidation of phenolic compounds and Maillard reactions [9,12,13,14,15]. In addition, Tao et al. [10] reported that chemical reactions affected by HHP are assumed to be promoted during the aging process according to Le Chatelier’s principle, which states that a decrease in volume induced by HHP could change the equilibrium of chemical reactions [16]. Altogether, this technique has a great potential in multiple fields, such as modifying wine composition, processing wines with low aging potential and reducing the sulfur dioxide additions during wine production.

In the past few years, there is a growing interest in multidisciplinary approaches, meaning the combination of microbial, physical and chemical treatments to elaborate high-quality low- or even free-SO_2_ wines [17]. Namely, due to multiple actions of SO_2_, antimicrobial and antioxidant, this additive is considered to be irreplaceable in wine production. However, in sensitive populations SO_2_ can cause allergic reactions and thus adversely affect health [18], so its use tends to be reduced. In the present literature, most studies regarding HHP-treated wines were carried out in either free-SO_2_ wines [11,12,13] or in wines with only one concentration of SO_2_ [9,13,14,19,20]. Recently, Christofi et al. [21] performed a study where the HHP treatment was studied in combination with different SO_2_ concentrations. However, there are no studies so far where the combination of HHP and different antioxidant treatments has been tested. The use of alternative physical and chemical treatments to SO_2_ in wine production was reviewed not so long ago by several authors [17,22,23]. These have investigated a lot of antioxidant and antimicrobial substitutes between which one of them is reduced glutathione (GSH). The addition of GSH, which has the ability to indirectly inhibit wine browning [24], preserve and improve aroma [25] and donate an electron to reactive oxygen species [26,27], has particularly increased the attention of many researchers. Although effective, so far, studied physical and chemical techniques do not possess the multiple SO_2_ action. Therefore, the aims of this paper were (i) to evaluate the effects of various HHP processing conditions on the phenolic and color composition of red and white wines right after the treatment and (ii) to investigate the potential use of HHP in winemaking along with the addition of antioxidants (glutathione and sulfur dioxide) during 12 months of aging.

## 2. Materials and Methods

### 2.1. Samples and Experimental Conditions

**Figure 1:**
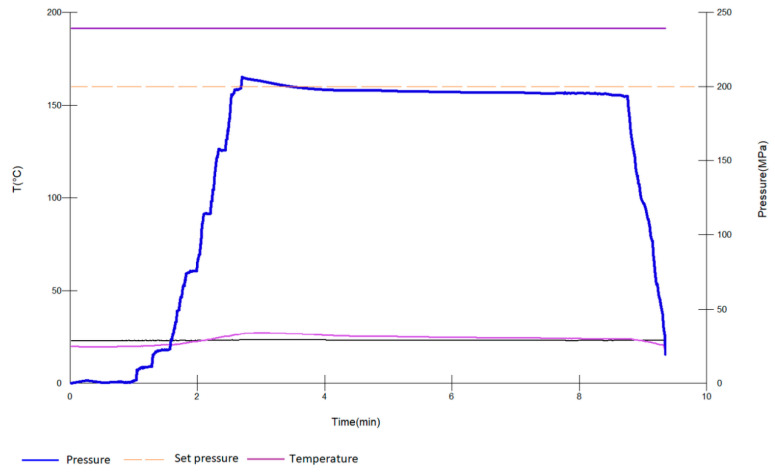
Example of the schematic diagram of the pressure and temperature during high hydrostatic pressure (HHP) processing of wines.

Red Cabernet Sauvignon and white Graševina wines with low SO_2_ content were produced by Erdutski vinogradi (Erdut, Croatia) during the 2016 harvest. For both red and white wines, classical winemaking procedures were used. In the red winemaking process, the Cabernet Sauvignon grapes were destemmed and gently crushed after being harvested and were placed in stainless steel tanks. Additionally, enzymes (3 mL/100 kg of Lafase XL Extraction, Laffort, France) and bisulfite solution (20 mg/L of total SO_2_) were added. Prior to the fermentation process, the must was inoculated with rehydrated yeast (20 g/hL of Zymaflore RX60^®^, Laffort, Bordeaux, France). The maceration/fermentation was carried out under 25 °C for 14 days. After 14 days of maceration, when alcoholic fermentation was finished, the wine was racked and pressed. Then, lactic acid bacteria (Lactoenos 450 PreAc^®^, Laffort, Bordeaux, France) were added to the wine for malolactic fermentation. After malolactic fermentation was over, wine was immediately decanted, microfiltered (0.2–0.4 μ) and sulfited at a concentration of 25 mg/L of free SO_2_. The basic parameters of red wine at the start of our experiment were: 13.1% *v*/*v* alcohol, pH 3.46, total acidity 5.3 g/L (as tartaric acid), volatile acidity 0.61 g/L (as acetic acid), reducing sugar 4.1 g/L, lactic acid 1.3 g/L, malic acid 0.1 g/L and dry extract 31.7 g/L. In the white winemaking process, after the Graševina grapes were immediately destemmed and crushed, reductive pressing with the addition of enzymes (2 mL/hL of Lafase XL Clarification, Laffort, France) and bisulfite solution (20 mg/L of total SO_2_) was carried out. After must was clarified by cold settling, it was transferred in stainless steel tanks for fermentation. Then, it was inoculated with rehydrated yeast (20 g/hL of Zymaflore X16^®^, Laffort, France). The fermentation conditions were as follows: temperature under 16 °C and duration of 12 days. After alcoholic fermentation, the wine was decanted, stabilized with 60 g/hL of Microcol^®^Alpha (Laffort, France), microfiltered (0.2 μ) and sulfited at a concentration of 25 mg/L of free SO_2_. The basic parameters of white wine at the start of our experiment were: 11.4% *v*/*v* alcohol, pH 3.37, total acidity 5.1 g/L (as tartaric acid), volatile acidity 0.31 g/L (as acetic acid), reducing sugar 2.8 g/L, lactic acid 0.3 g/L, malic acid 1.2 g/L and dry extract 20.2 g/L. These conventional wine analyses were carried out according to the official methods OIV-MA-AS312-01B, OIV-MA-AS313-15, OIV-MA-AS313-01, OIV-MA-AS313-02, OIV-MA-AS311-01A, OIV-MA-AS313-07, OIV-MA-AS313-10, and OIV-MA-AS2-03A of the International Organization of Vine and Wine [28]. Prior to pressurization, both red and white wines were first bottled in 100 mL plastic bottles and vacuum-sealed using plastic bags. The samples were further transferred to the pressure chamber of the high hydrostatic pressure system (Stansted Fluid Power FPG7100, Harlow, UK). Propylene glycol was used as the pressure-transmitting medium. Wine samples were pressurized during 5, 15 and 25 min at 200, 400 and 600 MPa. Nonthermal conditions were maintained during HHP processing with a maximum temperature ≤ 25 °C. All treatments were carried out in triplicate. The wines’ color and phenolic composition were analyzed immediately after performed pressurization. Besides that, the analyses of physicochemical parameters such as dissolved oxygen, total and free SO_2_ were also performed and have already presented in our previous work [29]. Namely, the dissolved oxygen was measured using a luminescent dissolved-oxygen sensor (NomaSense™ O_2_ P6000, Nomacorc, Belgium), and the obtained values ranged from 1.2 to 1.9 mg/L in control wines and 1.9 to 2.6 mg/L in pressurized red and white wines, respectively. As already presented in our previous work, the total and free SO_2_ were also determined by potentiometry using a sulfur dioxide measurement device (LDS Sulfilyser, Laboratories Dujardin-Salleron, Noizay, France), and the results showed 20 and 10 mg/L of total and free SO_2_ in pressurized and control red wines, as well as 70 and 25 mg/L of total and free SO_2_ in pressurized and control white wines, respectively [29]. Figure 1 shows a schematic diagram of experimental variables applied during HHP treatment of the wines.

### 2.2. HHP Treatment and Antioxidants—Wine Bottle Aging

In order to investigate the effect of HHP treatment in combination with SO_2_ and GSH additions on wine color, phenolic and aroma profile during 12 months of aging in bottles, the following red and white wines were used: standard SO_2_ wine (25 mg/L free SO_2_—red wine; 45 mg/L free SO_2_—white wine), low SO_2_+GSH wine (10 mg/L free SO_2_+20 mg/L GSH—red wine; 25 mg/L free SO_2_+20 mg/L GSH—white wine) and low SO_2_ wine (10 mg/L free SO_2_—red wine; 25 mg/L free SO_2_—white wine). Control samples were the standard SO_2_ wines not subjected to HHP treatment. An HHP of 200 MPa for 5 min was used, as this treatment resulted in similar or even slightly improved phenolic profile of pressurized wines compared to control (untreated) wines established in the first phase of the experiment as described in the Section 2.1. Additionally, it was reported that HHP in the range of 200–500 MPa can offer adequate inactivation rate of bacteria and yeasts in red and white wines, suggesting that it may be used to produce microbiologically stable wines [30]. All treatments were run in triplicate. After HHP processing, all wines (pressurized and unpressurized) were sealed in glass wine bottles and stored at 12 °C for 12 months. The chemical analyses (color, phenolic and aroma composition) were conducted on each wine after 0, 3, 6 and 12 months of aging in bottles.

As already presented in our previous work [29], the concentrations of dissolved oxygen, total and free SO_2_ were also controlled at the point of bottling and during 12 months of aging. As previously mentioned in Section 2.1., we used the same analytical methods and the results showed that the initial levels of dissolved oxygen at bottling in red wine amounted up to 2.2 mg/L in both pressurized and control wines, while after 12 months the levels were in the range from 0.4 to 0.6 mg/L. In the case of white wine, the initial levels were around 1.1 and 1.4 mg/L in control and pressurized samples, while at the end of aging the levels amounted up to 0.3 and 0.6 mg/L in control and pressurized samples, respectively [29]. During aging, pressurized standard SO2 wines (red and white) showed similar or slightly lower levels of total and free SO_2_ compared to untreated ones, amounting around 20 and 15 mg/L in the red wine and 80 and 25 mg/L in the white wine after 12 months. Also, the standard SO_2_ wines were characterized by lower amounts of dissolved oxygen, whereas the addition of GSH had no significant effect on oxygen and SO_2_ consumption rate in the red and white wines [29].

In exception, the volatile acidity (as acetic acid), known as important marker of microbiological spoilage, was monitored in this phase of experiment in order to assess the final quality of the wines. This parameter was analyzed according to the official OIV method [28]. After 12 months of aging, the data related to the volatile acidity showed the concentrations for red wine up to 0.69 g/L in control and in the range from 0.71 to 0.76 g/L in pressurized samples, and for white wine up to 0.37 g/L in control and from 0.43 to 0.47 g/L in pressurized samples, respectively. The obtained values were below the maximum allowable concentration of acetic acid in wines, which amounts to approximately around 1 g/L.

### 2.3. Chemical Analysis of Wine

Color properties (lightness, redness/greenness, yellowness/blueness, chroma, hue angle and total color difference) of the wine samples were determined using the CIELab system according to the OIV method [28]. Total phenolics (TP) were determined according to Singleton and Rossi [31], total anthocyanins (TA) according to Ribéreau-Gayon and Stonestreet [32] and total tannins (TT) according to Ribéreau-Gayon and Stonestreet [33].

Changes in phenolic composition of the red wine were monitored by high-performance liquid chromatography (HPLC). Analysis of free anthocyanins (FA) (delphinidin (Dph), cyanidin (Cy), petunidin (Pt), peonidin (Pn) and malvidin (Mv) -3-*O*-glucosides; peonidin- and malvidin-3-*O*-glucoside acetate (PnAc and MvAc); peonidin- and malvidin-3-*O*-glucoside *p*-coumarates (PnCm and MvCm)) was conducted according to the method described by Lorrain et al. [34]. The separation was performed on a Phenomenex Nucleosil C18 (250 mm × 4.6 mm, 5 μm) column and the mobile phases were water/formic acid (95:5, *v*/*v*) and acetonitrile/formic acid (95:5, *v*/*v*). The mobile phase gradient was: 0–25 min, 10–35% B linear; 25–26 min, 35–100% B linear; 26–28 min, 100% B isocratic; 28–29 min, 100–10% B linear, with re-equilibration of the column from 29–35 min under initial gradient conditions. The analysis conditions were: injection volume 20 μL, column temperature 40 °C, flow rate 1 mL/min and detection wavelength 520 nm. Results are expressed as the sum of free anthocyanins [35]. Analysis of individual flavanols (Fl) ((+)-catechin, (-)-epicatechin, procyanidins B1, B2, B3, B4 and C1) was conducted according to Ćurko et al. [36]. The separation was performed on a LiChrospher RP-18 (250 mm × 4 mm, 5 µm) column. The injected volume was also 20 μL. The mobile phase consisted of two solvents: water/formic acid (99:1, *v*/*v*) and acetonitrile/formic acid (99:1, *v*/*v*). The gradient conditions were: 0–11 min, 3–8% B linear; 11–16 min, 8% B isocratic; 16–20 min, 8–10% B linear; 20–27 min, 10% B isocratic; 27–32 min, 10–12% B linear; 32–34 min, 12–14% B linear; 34–45 min, 14–25% B linear; 45–46 min, 25–100% B linear; 46–50 min, 100% B isocratic, 50–51 min, 100–3% B linear, with re-equilibration of the column from 51–55 min under initial gradient conditions. The flow rate was 1 mL/min, column temperature 25 °C and detection wavelengths were 280 nm (excitation) and 320 nm (emission). Results are expressed as the sum of flavanols [35].

Changes in phenolic composition of the white wine were monitored by HPLC analysis of phenolic acids (Pa) (hydroxybenzoic (gallic, protocatechuic, vanillic and syringic) and hydroxycinnamic (caftaric, chlorogenic, caffeic, *p*-coumaric and ferulic)) and individual flavanols (Fl) ((+)-catechin, (-)-epicatechin, procyanidins B1 and B2) according to the method described by Monagas et al. [37].

For the phenolic acids and flavanols analysis, a column Phenomenex Gemini C18 (250 mm × 4.6 mm, 5 μm) was used. A modified gradient consisting of water/formic acid (98:2, *v*/*v*) and methanol was applied at flow rate of 1 mL/min as follows: 0 min, 2% B; 20 min, 32% B; 30 min, 40% B; 40–50 min, 50% B; 53 min, 2% B, with re-equilibration of the column from 53–55 min under initial gradient conditions. This simultaneous separation was conducted under following conditions: column temperature 25 °C, injection volume 20 μL and detection wavelengths 280 nm (hydroxybenzoic acids and flavanols) and 320 nm (hydroxycinnamic acids). Results are expressed as the sum of phenolic acids and sum of flavanols [38].

The aroma profile of the red and white wine samples was characterized by gas chromatographic analysis in detail described by Tomašević et al. [39]. Solid-phase microextraction (SPME) and gas chromatography-mass spectrometry (GC-MS) were used to extract and analyze free aroma compounds. For SPME extraction, 10 mL of wine sample, containing internal standard n-amyl alcohol (20 mg/L), was placed in the vial containing NaCl p.a. (2 g) and sealed with a crimp cap and silicone-PTFE septum. After the 100 μm PDMS fiber (Supelco, Bellefonte, USA) was exposed in the upper space of the vial at 40 °C for 30 min with constant shaking, it was immediately transferred to the GC injector for desorption at 250 °C for 5 min in splitless mode. Additionally, chromatographic analysis was performed on BP20 capillary (50 m × 220 μm × 0.25 μm) column (SGE Analytical Science, Victoria, Australia). The GC-MS working conditions were as follows: the detector interface temperature 250 °C, the electron ionization ion source at 70 eV and 280 °C, vector gas helium 5.0 and constant flow rate 1.2 mL/min. The temperature program for aroma analysis was: 40 °C, 5 min → 200 °C, 3 °C/min → 240 °C, 30 °C/min; 1 min, with the acquisition in scan mode. Due to a large number of identified and quantified aroma compounds, they were classified into four aroma groups: esters (*i*-butyl acetate, *i*-amyl acetate, ethyl acetate, 2-phenylethyl acetate, hexyl acetate, ethyl butyrate, ethyl hexanoate, ethyl octanoate, ethyl decanoate and diethyl succinate), higher alcohols (amyl alcohol, phenylethyl alcohol, 1-hexanol and *cis*-3-hexenol), fatty acids (hexanoic, octanoic and decanoic acid) and terpenes (α-terpineol and linalool). Results are expressed as the sum of esters, sum of higher alcohols, sum of fatty acids and sum of terpenes.

### 2.4. Sensory Evaluation

The wines were subjected to sensory evaluation by the nine-point hedonic scale method, with 25 judges. Generally, each sample (25–30 mL of wine) was presented in a coded, standard ISO 3591 tasting wineglass covered with a plastic Petri dish and served randomly. The judges were required to evaluate the treated wines with respect to the control (untreated). Additionally, all judges were informed that the wines had undergone different treatments, but they did not have any details of the experimental design. The total effect of combined HHP and antioxidant treatments on color, odor and taste was evaluated with a verbal scale of 9 possible responses (1 = dislike extremely, 2 = dislike very much, 3 = dislike moderately, 4 = dislike slightly, 5 = neither like nor dislike, 6 = like slightly, 7 = like moderately, 8 = like very much, 9 = like extremely) [40]. The sensory analysis was performed on each wine after 0, 3, 6 and 12 months of bottle aging. Mean liking ratings and standard deviations were calculated.

### 2.5. Statistical Analysis

The statistical analysis was performed by one-way ANOVA in Statistica V.10 software (StatSoft, Tulsa, OK, USA). Tukey’s and Duncan’s tests were used as a comparison test when samples were significantly different after ANOVA (*p* < 0.05) for chemical and sensory analysis. The data were expressed as the mean value of three analytical repetitions with standard deviation.

## 3. Results and Discussion

### 3.1. Phenolic Profile and Color Properties of Red and White Wines after HHP Processing

The profiles for total and individual phenolic compounds, as well as color properties in both HHP-treated and untreated red and white wines are provided in Table 1; Table 2, respectively.

#### 3.1.1. Red Wine

As can be seen in Table 1, slight changes occurred in the phenolic profile and color properties of the red wine after HHP processing. Generally, applied HHP treatments resulted in slightly lower content of TP and TA, except TT which remained constant. Also, the results concerning the evaluation of the sum of FA and sum of Fl showed that the content of individual anthocyanins and flavanols slightly decreased in all pressurized wines compared to control. These changes, although statistically significant (*p* < 0.05), remained relatively small at lower pressure levels. Almost no significant differences were observed between the sample pressurized at 200 MPa for 5 min and untreated (control) wine, respectively. Nevertheless, the main variations obtained can related to the differences in pressures applied, indicating that this parameter was a more discriminatory factor than the processing time. Namely, a decrease in both total and individual phenolics was most pronounced in samples treated with higher pressure (600 MPa). Moreover, when considering only the effect of pressurization time, longer treatments also resulted in a lower content of analyzed phenolic compounds. Taken all together, an HHP treatment of 600 MPa for 25 min resulted in the most significant decrease of all phenolics in the red wine when compared to the unpressurized sample. These results are in accordance with the findings of Tao et al. [41], who reported that pressurization at conditions of 250–650 MPa for 15–120 min mainly resulted in the decrease of phenolic compounds such as total phenolics, total anthocyanins, flavonols, tannins and tartaric esters. The same study also demonstrated the significant impact of both process parameters, pressure and time, on wine quality, where the first had the more influence than the latter. Chen et al. [42] also observed the decrease in the content of flavanols and the increase in phenolic acids of young red wine after HHP treatments (100–600 MPa/30 min, 500 MPa/5–60 min).

Furthermore, pressurized red wine samples showed different values (*p* < 0.05) of the CIELab parameters, when compared with the unpressurized ones (Table 1), respectively. A slight increase in parameters L*, a*, b*, C* and H^*^ was observed after HHP treatments, indicating a slight change in the wine color, shifting from red-purple to more orange-red and lighter. The observed changes were particularly pronounced after applying higher pressure (600 MPa) and longer time of processing (25 min). Similarly, Sun et al. [43] reported significant changes of color properties (chroma and hue values) of young red wine after HHP treatments (100–600 MPa/30 min, 500 MPa/5–60 min). Furthermore, the total color differences (∆E*) between pressurized and unpressurized samples were calculated in order to determine whether the observed changes in the chromatic properties of the red wine were visually relevant. Generally, the values above 3 reflect differences which are noticeable and clearly perceived by the observer in the case of the red wine [44].The results demonstrated that ∆E^*^ values were even lower than 1 or around 1 CIELab unit after HHP treatments at 200 and 400 MPa during 5, 15 and 25 min. However, all treatments at 600 MPa resulted in ∆E^*^ values around 4 CIELab units, which is clearly higher than limit value of 3 CIELab units considered for perceiving the differences by the human eye in red wine [44].

#### 3.1.2. White Wine

The results regarding the phenolic and color changes of white wine after HHP treatments also showed significant differences (*p* < 0.05) between pressurized and unpressurized samples (Table 2), respectively. As it can be observed, the pressurized wines were characterized by slightly lower content of TP, sum of Pa and sum of Fl. In addition, most of individual phenolic acids (vanillic, syringic, caftaric, chlorogenic and caffeic acid) and flavanols showed decreasing trend after applying higher HHP process conditions (pressure and time). But, on the other hand, slightly higher content of gallic, protocatechuic and *p*-coumaric acid was found in HHP-treated samples at lower pressures of 200 and 400 MPa compared to control wine, while after applying pressure of 600 MPa differences diminished. Particularly, the pressurized sample at 200 MPa for 5 min compared to control wine was significantly higher in content of previously mentioned phenolic acids and additionally in chlorogenic and caffeic acid. On the other hand, the content of ferulic acid remain unchanged in all wines. This increasing trend in the content of corresponding phenolic acids could be explained by the possibility of pressure to promote the decomposition of some compounds [42]. Overall, as already observed in the case of red wine, the lowest content of analyzed phenolic compounds in the white wine was also determined after treatment at 600 MPa for 25 min. Similar results were reported by Briones-Labarca et al. [19], whose study showed that the total phenolic and flavonoid contents of young white wine were not severely reduced by HHP treatments (300–500 MPa/5–15 min). Moreover, Santos et al. [12] found that HHP treatments (425 and 500 MPa for 5 min) had no effect on the total phenolics and antioxidant activity of white wine immediately after processing.

As regards the white wine color after HHP treatments, most of the pressurized samples were characterized by slightly lower values of L* and C* and higher values of a* and b* (*p* < 0.05) compared to control wine, respectively. The observed changes indicate that the wine color shifts from pale and practically colorless to a more yellow color. However, all samples treated at 600 MPa showed oppositely significantly lower values of parameters a* and H*, while values of b* and C* were very close to ones of control. In all other cases, there was no significant difference in parameter H^*^ when comparing with unpressurized sample. Moreover, the pressurized sample at 200 MPa for 5 min and the unpressurized one did not differ drastically in their color parameters compared to all other samples. Additionally, the total color difference values (∆E*) indicated that the pressurized wines at the highest pressure level (600 MPa) visually differed from the control sample, since the values were higher than 3 CIELab units (3.7 and 3.9, respectively). On the other hand, all other HHP treatments led to wines more like the untreated one. Namely, established ∆E* values were in the 0.7–2.0 CIELab unit range, which cannot be clearly detected by the human eye. These results agree with those reported by Briones-Labarca et al. [19], who observed slight changes in the chromatic properties of white wine after applying HHP, but also stating that these changes were not visually perceived.

In addition, our own earlier work demonstrated that, in general, these HHP processing conditions resulted in slight aroma changes, primarily decrease of volatiles like esters, fatty acids and terpenes, and increase of higher alcohols in both red and white wine [45]. Also, it was found that these changes were more pronounced in red wine, and particularly after pressurization with higher pressure of 600 MPa and longer processing time of 25 min. As regards to the above-mentioned, with properly selected treatment conditions not causing major quality changes, HHP could be very promising in wine technology to complement the protective action of SO_2_, enabling to reduce its content in wines.

### 3.2. Phenolic and Aroma Changes of Red Wine during 12 Months of Aging Induced by HHP and Antioxidant Treatments

#### 3.2.1. Phenolic Profile and Color Properties

**Figure 2:**
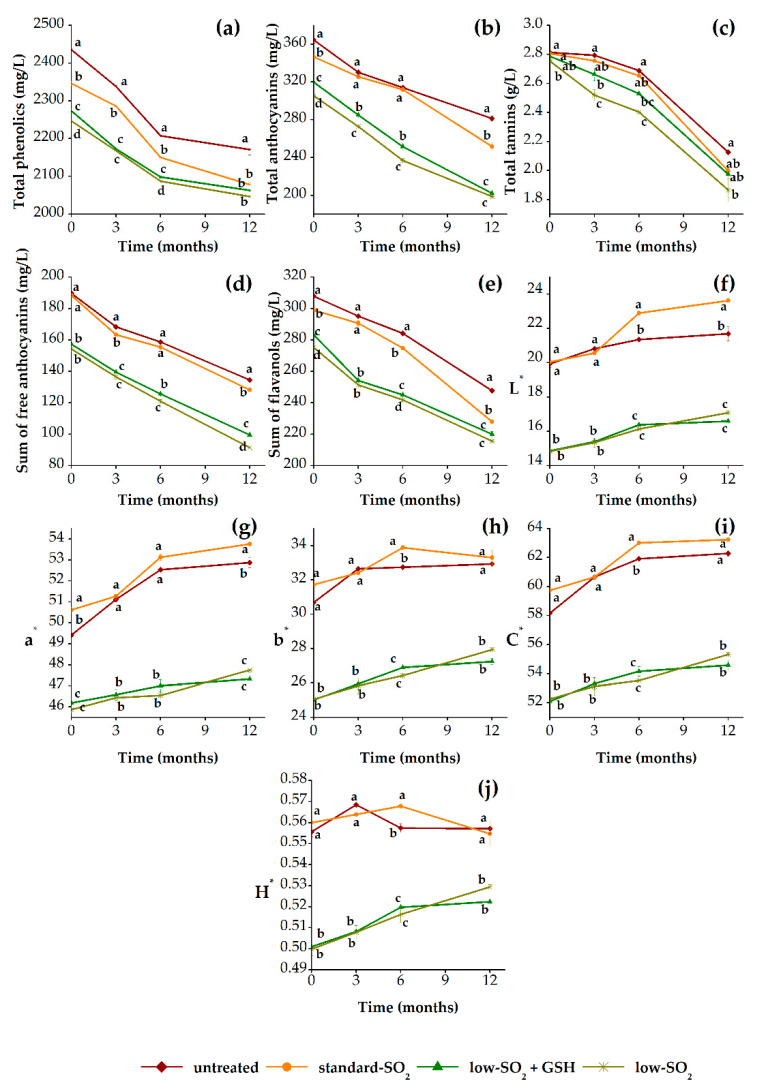
Phenolic (**a**–**e**) and color (**f**–**j**) changes of pressurized (standard SO_2_, low SO_2_+GSH and low SO_2_) and unpressurized (untreated) red wine samples during 12 months of aging in bottles. GSH: glutathione.

Figure 2 presents the evolution of total phenolics (TP), total anthocyanins (TA), total tannins (TT), sum of free anthocyanins (FA), sum of flavanols (Fl) and color properties (L*, a*, b*, C* and H*) of HHP-treated and untreated red wine samples during 12 months of aging in relation to their antioxidants (SO_2_ and GSH) content. In general, there is a decreasing trend in the content of analyzed phenolics with time, independently of applied treatments. The phenolic changes during aging of wine are mainly due to their potential chemical oxidation, polymerization, condensation and precipitation [46,47]. As can be seen, the significant differences (*p* < 0.05) were observed between phenolic composition of pressurized and unpressurized wines along the aging period, respectively (Figure 2a–e). At the beginning of aging, pressurized samples contained lower concentrations of TP, TA and Fl when compared with control (untreated) wine. However, the HHP effect on the content of FA and TT was not observed immediately after pressurization. These differences slightly varied between pressurized and unpressurized samples during first 6 months, but after 12 months of aging significant differences can be clearly seen in TP, TA, FA and Fl, while there were no major changes in TT content. These agrees with the findings of other studies [9,11,20,21,48] which demonstrate that HHP treatment results in a decrease of phenolic compounds, primarily anthocyanins and flavanols. HHP-induced changes can be related to the reduction in volume during HHP processing, which could impact the chemical equilibrium of a reaction [30]. These results support the hypothesis that pressurization reduces the content of anthocyanins and flavanols due to enhancement of numerous chemical reactions involving phenolic compound such as condensation, polymerization and oxidation [21]. In addition, the lowest content of analyzed phenolic compounds among pressurized samples was observed in the sample with low SO_2_ content. Obviously, the HHP treatment in combination with higher content of SO_2_ can slow down the chemical reactions rate, which are otherwise accelerated in the treated samples with higher concentrations of antioxidants. However, light effect of GSH on phenolic composition was evident at the beginning of aging and up to a period of 6 months, but after 12 months no differences were found between low-SO_2_+GSH and low-SO_2_ wines (except in FA). Therefore, the different trends observed among treated samples are not just a consequence of potential acceleration of chemical reactions by HHP, but also, they are the result of different SO_2_ content in wines. Namely, the SO_2_ actions in wine primarily refer to the reduction of polymerization reactions rate of phenolic compounds and the protection from oxidation [9,11,21]. However, pressurized standard-SO_2_ and low-SO_2_+GSH samples showed similar content of TP and TT after 12 months of aging. As far as we are aware, this is the first time that the HHP treatment was investigated in combination with the addition of different amounts of antioxidants, SO_2_ and GSH. Nevertheless, few earlier studies have focused on the joint effects of HHP and SO_2_. For instance, the study by Santos et al. [11] compared the pressurized and unpressurized wine samples containing 0 and 40 mg/L of SO_2_. Recently, the study of Christofi et al. [21] involved pressurized and unpressurized red wine samples containing 0, 30, 60 and 100 mg/L of SO_2_. The same authors found that a combination of HHP treatment (350 MPa/10 min) and 60 mg/L SO_2_ may slow down the rate of chemical reactions, which take place much faster in pressurized samples.

Since the phenolic composition and color of wine are closely related, it is also important to highlight the results regarding color properties (Figure 2f–j). In accordance with the findings of other research [9,11,21,48], an increase of all color parameters, namely L*, a*, b*, C* and H*, with time was observed in all wine samples. It is known that anthocyanins provide the initial color of red wine, while as wine ages, its color significantly changes due to the decrease of free anthocyanins and formation of polymeric pigments [30,49]. This increment of corresponding color parameters indicates that the color becomes more lighter and orange-red-like in the aged wines [9,15]. Furthermore, no significant differences were observed in color parameters among pressurized and unpressurized standard-SO_2_ wines up to the period of 3 months of aging. However, after 12 months of aging, significant differences (*p* < 0.05) in parameters L^*^ and a^*^ were found, whereas parameters b^*^, C^*^ and H^*^ remained unchanged. Moreover, the pressurized standard-SO_2_ sample presented higher values of CIELab parameters when compared with control wine after 12 months of aging, respectively. Further, there was obvious difference among HHP-treated samples concerning the effect of antioxidants, primarily SO_2_, while no GSH effect was noticed. The samples containing lower content of SO_2_ as well as GSH presented the same trends and values of color parameters during observed period of aging. These wines had much lower values of CIELab parameters compared to samples with standard SO_2_ content (both pressurized and unpressurized). A study by van Wyk et al. [20] also found that HHP treatment (400 MPa/5 s) resulted in decreased color density and increased brownish color in SO_2_ free red wine. Moreover, the total color difference (∆E^*^) was calculated to express the overall color difference between treated samples and control. In the early stages after pressurization and after 12 months of aging, ∆E^*^ values for the pressurized standard-SO_2_ wine were lower than 3 CIELab units, increasing along the aging time from 0.4 to 2.2 (data not shown). These results suggest that the difference in color of the pressurized standard-SO_2_ sample in relation to the unpressurized (control) wine was not perceived by the human eye. This seems to be due to protective effect of SO_2_, which can protect wine from excessive oxidation of phenolic compounds and consequently avoid the undesirable modifications [50]. On the other hand, for the rest of pressurized samples (low SO_2_+GSH and low SO_2_) ∆E^*^ values were around 8 at the beginning of aging, when compared to control. Moreover, at the end of 12 months of aging, ∆E^*^ values increased to around 9 and 10, respectively. These results indicate that the color changes are mainly due to a combination of HHP treatment with different content of SO_2_ in presented wines.

#### 3.2.2. Aroma Profile

**Figure 3:**
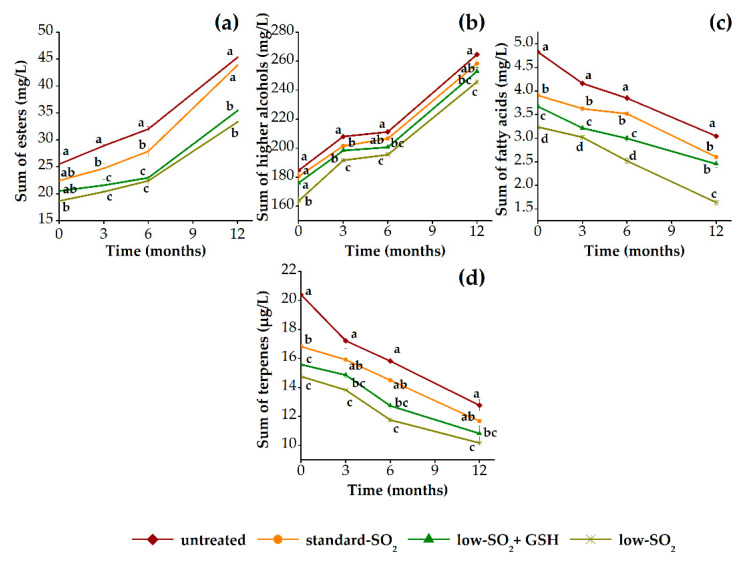
Aroma changes of pressurized (standard SO_2_, low SO_2_+GSH and low SO_2_) and unpressurized (untreated) red wine samples during 12 months of aging in bottles: (**a**) sum of esters; (**b**) sum of higher alcohols; (**c**) sum of fatty acids; (**d**) sum of terpenes. GSH: glutathione.

Figure 3 shows the evolution of sum of esters, sum of higher alcohols, sum of fatty acids and sum of terpenes of HHP-treated and untreated red wine samples during 12 months of aging in relation to their content of antioxidants (SO_2_ and GSH). Generally, the content of esters and higher alcohols increased, while a decrease in the content of fatty acids and terpenes was observed in all wines during aging period of 12 months (Figure 3a–d). However, observed slight increase of esters is due to increase in the content of two individual aroma compounds, namely ethyl acetate and diethyl succinate, while other quantified compounds included in sum of esters actually decreased. Esters are reported to decrease during aging due to chemical reactions of hydrolysis or oxidation. The same evolution pattern follows terpenes, which also decrease during aging [51]. Altogether, in this way, the wines are known to lose some of their fruity and floral aromas. Furthermore, higher alcohols are reported to be mainly stable during aging, but some increases have been observed, which are explained through hydrolysis of the corresponding esters [52] or a certain microbial activity occurred in wines [39]. On the other hand, the stability of fatty acids is not uniform, as some compounds could increase while others decrease or remain stable during aging [51]. As can be seen from Figure 3, HHP-treated samples contained, in general, slightly lower content of aroma compounds when compared with the untreated sample. This can be due to an increase of interactions among aroma and phenolic compounds in wine during aging induced by HHP [13]. Immediately after pressurization, no differences were found in the content of esters and higher alcohols among unpressurized and pressurized samples, whereas the significant differences (*p* < 0.05) were observed for the content of fatty acids and terpenes, respectively. Up to a period of 3 months of aging, the differences were significant for almost all aroma groups (except terpenes), while after 6 months they were noticeable in the case of esters and fatty acids. Additionally, after 12 months of aging, significant differences were determined only for the content of fatty acids, indicating that HHP treatment influenced this group of aroma compounds. Although the aroma is an important factor in defining the quality of wine, in the present literature there is only one study that specifically determined the aroma composition of HHP-treated red wine along the storage period [13], while all other studies were primarily oriented toward the effect of HHP on wine sensory attributes [9,11,20,21]. Namely, Santos et al. [13] demonstrated that there were minor differences in aroma composition of pressurized wines after 2 months of storage, while after 9 months quite remarkable changes occurred, indicating a significant impact of HHP on aroma composition of SO_2_ free red and white wines. Also, the same authors found that treated samples contained higher content of aldehydes, ketones, acetals and furans, and suggested that HHP treatment accelerates oxidation of higher alcohols and fatty acids and Maillard reactions, lastly giving the aroma profile of aged wines. Further, regarding the effect of SO_2_ content, it can be clearly seen that among treated samples those with low SO_2_ content were characterized by lower content of all aroma compounds, respectively. Probably, the well-known antioxidant activity of SO_2_ resulted in its inhibitory action of slowing down their loss during aging. It was already presented that the presence or absence of SO_2_ had a great impact on the evolution of esters and higher alcohols and to lesser extent fatty acids during wine aging in the bottle [53]. Aside from that, not of lesser importance is the effect of GSH, which had a significant impact on fatty acids and much less impact on higher alcohols and terpenes, while no effect was observed for the group of esters during aging period. As regards to the role of GSH in protecting wine aroma compounds, it was shown that this reduced form of glutathione can react as a strong nucleophile with quinones [54]. Additionally, after 12 months of aging, the pressurized wines (standard SO_2_ and low SO_2_+GSH) showed very close values in the most of aroma compounds, except esters, indicating that HHP can be applied with lower content of SO_2_ without causing major changes in aroma composition.

### 3.3. Phenolic and Aroma Changes of White Wine during 12 Months of Aging Induced by HHP and Antioxidant Treatments

#### 3.3.1. Phenolic Profile and Color Properties

**Figure 4:**
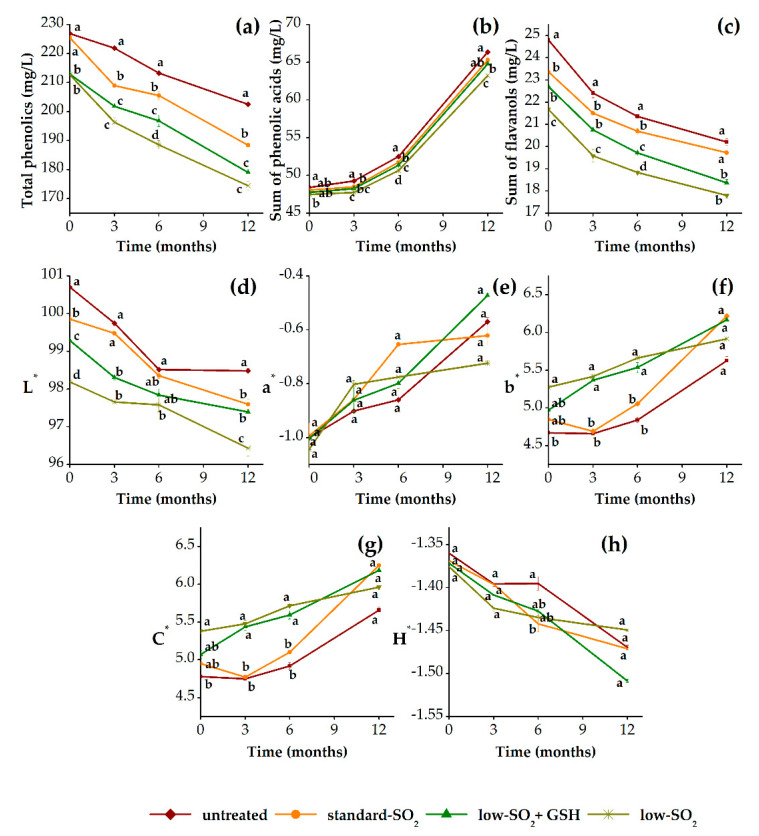
Phenolic (**a**–**c**) and color (**d**–**h**) changes of pressurized (standard SO_2_, low SO_2_+GSH and low SO_2_) and unpressurized (untreated) white wine samples during 12 months of aging in bottles. GSH: glutathione.

Figure 4 presents the evolution of total phenolics (TP), sum of phenolic acids (Pa), sum of flavanols (Fl) and color properties (L*, a*, b*, C* and H*) of HHP-treated and untreated white wine samples during 12 months of aging in relation to their antioxidant (SO_2_ and GSH) content. As it can be observed, the content of TP and Fl decreased, while the content of Pa increased with aging time in all presented samples (Figure 4a–c). Generally, during aging of white wine, browning and oxidation reactions take place. The most important phenolic compounds involved in these reactions are hydroxycinnamic esters and flavanols, which content consequently decreases with time [55]. On the other hand, this reduction of hydroxycinnamates due to hydrolysis reactions is mainly responsible for the increment of certain free phenolic acids [56]. Additionally, this increase can be related to their participation in reactions with glutathione [57]. Moreover, the pressurized samples were characterized by slightly lower content of analyzed phenolics compared to control wine. In general, at the beginning of aging, no significant differences were observed in the content of TP and Pa (except Fl) between pressurized and unpressurized standard-SO_2_ wines, respectively. However, after 3 months and up to a period of 6 months of aging, significant differences (*p* < 0.05) were observed among pressurized and unpressurized standard-SO_2_ wines in overall phenolic composition. At the end of the aging period of 12 months, HHP treatment significantly influenced the content of TP, whereas no significant differences in Pa and Fl content compared to control were found. Santos et al. [12] also observed the decrease of the total phenolic content as well as antioxidant activity in pressurized white wine samples after 12 months of storage. It is suggested that the generation of highly reactive radicals during HHP processing and enhancement of oxidation and polymerization reactions of phenolic compounds are responsible for reduction in their content [9,12,19,42]. Concerning the effect of antioxidant treatments (SO_2_ and GSH), the pressurized low SO_2_ wine showed the lowest content of analyzed phenolics. Namely, higher content of SO_2_ seems to obstruct the loss of these compounds during aging due to the reasons described earlier. Although, after 6 months of aging, GSH effect was evident in TP, Pa and Fl, respectively, after 12 months, it was only noticed for Pa. Since all phenolic compounds are susceptible to oxidation changes, GSH could react with the quinonic form of the hydroxycinnamic acids through an electrophilic addition, triggering the regeneration of free forms [58]. Additionally, the pressurized wines, standard SO_2_ and low SO_2_+GSH, presented very similar values in Pa content at the end of aging.

Considering the color properties of the white wine, in general, there was a decreasing trend in parameters L* and H*, while parameters a*, b* and C* increased with time in all wine samples (Figure 4d–h). During aging, oxidative processes involving phenolics would surely result in a change of color, from pale yellow to more yellow-brown. Other authors also reported similar changes in the chromatic data during aging of white wine [12,56,59], where oxidation of phenolics, especially flavanols (catechins and procyanidins) to quinones, which than polymerize to form yellow-brown products, are mainly responsible for these color changes. In addition, no significant differences were found in the most of the CIELab parameters, except lightness (L*), immediately after HHP treatment and during 12 months of aging between pressurized and unpressurized standard-SO_2_ wines. Furthermore, among pressurized wines, at the beginning of aging and after 12 months, there was only significant difference in parameter L*, whereas the values of parameters a*, b*, C* and H* did not differ significantly. However, there were some apparent differences in parameters L*, b* and C* between pressurized wines with standard SO_2_ and those with low SO_2_/low SO_2_+GSH content after 3 and 6 months of aging. A previous study by Santos et al. [12] showed that HHP-treated white wine without SO_2_ had more brownish color and lower phenolic content than untreated wines with 0 and 40 mg/L of SO_2_ after 12 months of bottle aging, indicating that HHP probably accelerates the Maillard reaction in white wine. Additionally, the results of calculated total color difference (∆E*) confirmed that the observed changes in the color parameters between pressurized and unpressurized samples were not visually relevant. Although, there is no specified limit value for determining that the color differences in white wine are observable by the human eye in the literature, all obtained values were far below 3 CIELab units (data not shown), otherwise considered as a relevant value in the case of red wine. During 12 months of aging, the pressurized standard-SO_2_ sample presented ∆E* values in the CIELab unit range from 0.3 to 1.1 in comparison to control. As emphasized earlier, SO_2_ is very important in preventing the oxidative color changes, particularly in white wines as they have less of other antioxidants such as phenolic compounds than the red wines. Furthermore, compared to control, pressurized low-SO_2_+GSH and low-SO_2_ wines showed slightly higher values of ∆E* ranging from 1.0 to 1.6 and from 1.2 to 2.6. Moreover, the addition of GSH in our case did not significantly affect the overall color of white wine, although it was reported that glutathione in the presence of small amounts of SO_2_ has the ability to delay the oxidative color changes and the formation of xanthylium cation pigments [60,61]. Therefore, combination of HHP treatment with the addition of antioxidants (SO_2_ and GSH) did not remarkably influence the color properties of white wine, except lightness, as stated above.

#### 3.3.2. Aroma Profile

**Figure 5:**
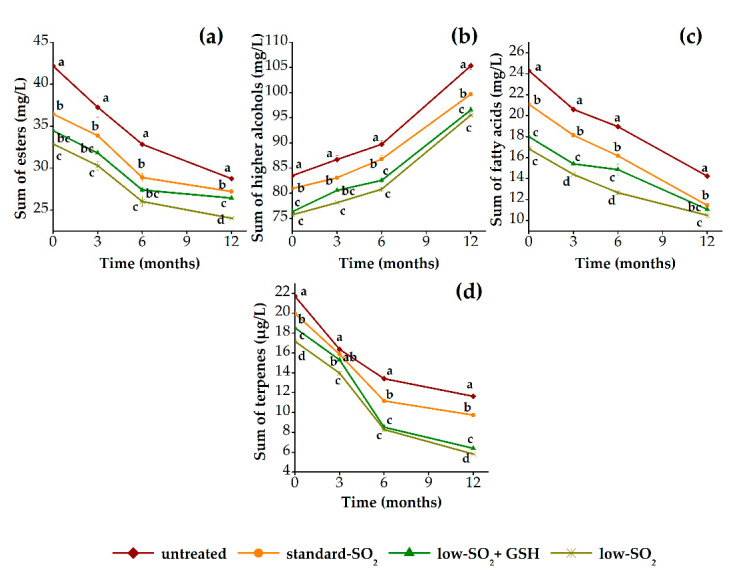
Aroma changes of pressurized (standard SO_2_, low SO_2_+GSH and low SO_2_) and unpressurized (untreated) white wine samples during 12 months of aging in bottles: (**a**) sum of esters; (**b**) sum of higher alcohols; (**c**) sum of fatty acids; (**d**) sum of terpenes. GSH: glutathione.

Figure 5 shows the evolution of sum of esters, sum of higher alcohols, sum of fatty acids and sum of terpenes of HHP-treated and untreated white wine samples during 12 months of aging in relation to their content of antioxidants (SO_2_ and GSH). The results showed that the content of esters, fatty acids and terpenes decreased, while the content of higher alcohols increased in all wines during 12 months of aging. These aroma changes are known to naturally occur during the wine aging process, as already described in the case of red wine. Namely, the transformation of aroma compounds leads to a loss of characteristic aromas of young wines and gradual formation of more complex aroma composition typical for aged wines [62]. In addition, significant difference (*p* < 0.05) between pressurized and unpressurized wines regarding their content of analyzed groups of aroma compounds were found at the beginning of aging and after 12 months (Figure 5a–d). Namely, the pressurized samples presented lower content of esters, higher alcohols, fatty acids and terpenes compared to control wine, respectively. Currently, there are only two studies that have investigated the effect of HHP and how it changes aroma composition as well as sensory properties of white wine during bottle aging [12,13]. As already described for the red wines, Santos et al. [13] found that the pressurized white wines were also characterized mainly by aldehydes, furans, acetals and ketones. The same authors explained that the higher content of ketones in pressurized wine is due to occurrence of oxidation of fatty acids with pressure. This observation explains the decrease in the content of fatty acids of HHP-treated wines discussed previously. Furthermore, both mentioned studies suggested that HHP treatment can accelerate the formation of wine aging aroma due to enhancement of Maillard reaction and fatty acid and alcohol oxidation. In relation to antioxidant treatments (SO_2_ and GSH), the significant differences were observed among pressurized wines in the content of aroma compounds during 12 months of aging. Particularly, the higher content of SO_2_ resulted in wines with higher content of all aroma groups, whereas no unique effect was found regarding the addition of GSH during the observed period of aging. The effects of SO_2_ on oxidation and aging of wine are well established [63,64,65]. Regarding wine aroma, it has been reported that SO_2_ protects several groups of aroma compounds, such as esters, higher alcohols and fatty acids, during aging of wine [66,67]. However, after 12 months, the GSH effect was noticeable on the content of esters and terpenes, while practically no effect was determined in the case of higher alcohols and fatty acids. The GSH, with its thiol group, can react as a strong nucleophile with quinones, and in this way protect important aroma compounds such as esters, terpenes and thiols [27]. Moreover, the addition of GSH in white wine production has been demonstrated to limit the accumulation of acetaldehyde and to preserve the aroma complexity and freshness after 12 months of bottle aging [68]. From these results it follows that from all HHP treatments performed, the combined HHP and standard-SO_2_ treatment reduced the rate of chemical reactions, such as hydrolysis or oxidation, to the greatest extent which seemed to happen faster in treated samples.

### 3.4. Sensory Changes of Red and White Wines during 12 Months of Aging Induced by HHP and Antioxidant Treatments

The sensory properties of wines were analyzed by the nine-point hedonic scale method to assess the organoleptic characteristics in terms of color, odor and taste. The influence of HHP treatment along with antioxidants addition (SO_2_ and GSH) on the wines’ sensory attributes with the results represented as the average scores of the panelists are shown in Table 3, for red and white wine, respectively. At the very beginning, the results showed that there were no significant differences among pressurized red wine samples for each of the attributes scored. On the other hand, in the case of white wine, there were significant differences (*p* < 0.05) between samples with higher and lower concentration of SO_2_ in terms of color and odor. After 3 months of aging, significant differences were found among standard- and low-SO_2_ white wines for each of the attributes, while in red wines the occurred differences were much less pronounced. A similar trend to that was observed after 6 months of aging. When sensory analysis was performed 12 months after bottling, very similar scores were given to standard SO_2_ and low SO_2_+GSH samples of both red and white wine, respectively (Table 3). In general, the lowest scores were assigned to both red and white wines with low SO_2_ content for each of the sensory attributes. Moreover, when comparing red and white wine, it can be seen that red wine samples had slightly higher ratings in all three analyzed attributes. Overall, after 12 months of bottle aging, both the treated red and white wines were evaluated with fairly good scores (7 = like moderately and 6 = like slightly). Generally, the degradation rate of aroma of red wines is slower compared to white wines due to a higher content of phenolic compounds, which have antioxidant properties. According to Fuhrman et al. [69], the limited antioxidant character of white wines makes them more susceptible to oxidation in contrast to red wines, which was probably the reason why combined HHP and antioxidant treatments affected the white wine sensory attributes slightly more than those of the red wine. Moreover, it seems that the changes in phenolic and aromatic composition induced by both HHP and antioxidant treatments, can modify the sensory quality of wines. However, the relationship between chemical composition and sensory attributes is not always easy to evaluate, due to the complexity of wine’s chemical composition and its numerous interacting components [70].

## 4. Conclusions

In this work, we first have investigated the influence of HHP treatments on red and white wines’ chemical composition. Besides that, the combination of HHP treatment and the addition of antioxidants (glutathione and sulfur dioxide) was examined on wine phenolic and aroma composition, as well as sensory properties during 12 months of bottle aging. The results of this study showed that slight changes occurred in the phenolic composition and color properties of red and white wines immediately after HHP treatments. In pressurized red wine these changes manifested as a decrease of both total and individual phenolic compounds, while all color parameters increased. Additionally, applied treatments resulted in the decrease of phenolic contents in white wine, with exception in the increase of some free phenolic acids. Regarding applied HHP conditions, higher pressures as well as longer processing times resulted in more noticeable changes of analyzed compounds, where the pressure was more responsible for main variations in data. After 12 months of aging, the HHP-treated red wines were characterized by lower content of TP, TA, FA and Fl, without major changes in the content of TT. On the other hand, HHP treatment after 12 months of aging did not influence most of the color parameters (except L* and a*) and aroma compounds (except fatty acids) of the red wine. Concerning the white wine, HHP treatment did not affect most of the phenolics (except TP) and color properties (except L*) after 12 months of aging, but it showed impact on the aroma compounds. Moreover, the higher content of antioxidants (SO_2_ and GSH) resulted in HHP-treated red and white wines with a higher content of analyzed phenolic and aroma compounds possibly due to the decreased rates of condensation and oxidation reactions. Namely, no significant differences were observed among pressurized standard-SO_2_ and low-SO_2_+GSH red wines in concentrations of aroma compounds, primarily fatty acids, while for the white wines this was mostly evident in the color properties. Finally, the sensory analysis also showed that the wine samples were distinguished primarily by different amounts of antioxidants used. Additionally, the effect of combined HHP and antioxidant treatments was slightly more pronounced in the white wine. Therefore, HHP should be considered as a potential alternative for complementing the antioxidant and antimicrobial actions of SO_2_. Thus, the aspect of multidisciplinary approaches such as the combination of physical and chemical treatments even with SO_2_ may help to reduce SO_2_ use during the wine production.

## Figures and Tables

**Table 1:** Phenolic profile and color parameters of pressurized and unpressurized red wine samples.

Analysis	RW	High Hydrostatic Pressure Processing								
	Untreated	200 MPa/5 min	200 MPa/15 min	200 MPa/25 min	400 MPa/5 min	400 MPa/15 min	400 MPa/25 min	600 MPa/5 min	600 MPa/15 min	600 MPa/25 min
TP (mg/L)	2455.0 ± 3.2 ^a^	2440.5 ± 4.5 ^ab^	2436.4 ± 6.4 ^bc^	2424.6 ± 5.1 ^cd^	2417.7 ± 0.6 ^d^	2396.4 ± 1.3 ^e^	2358.2 ± 3.9 ^f^	2366.8 ± 3.2 ^f^	2364.6 ± 3.9 ^f^	2337.3 ± 3.9 ^g^
TA (mg/L)	333.1 ± 0.4 ^a^	331.9 ± 2.9 ^ab^	328.6 ± 0.7 ^abc^	326.0 ± 2.1 ^bc^	330.4 ± 0.4 ^abc^	329.2 ± 0.4 ^abc^	326.4 ± 0.9 ^bc^	324.6 ± 2.2 ^c^	315.7 ± 2.5 ^d^	315.6 ± 1.6 ^d^
TT (g/L)	2.94 ± 0.06 ^a^	2.94 ± 0.06 ^a^	2.92 ± 0.07 ^a^	2.92 ± 0.06 ^a^	2.94 ± 0.02 ^a^	2.91 ± 0.11 ^a^	2.91 ± 0.00 ^a^	2.88 ± 0.05 ^a^	2.86 ± 0.03 ^a^	2.85 ± 0.05 ^a^
FA (mg/L)										
Dph	18.42 ± 0.34 ^a^	17.92 ± 0.09 ^ab^	18.20 ± 0.15 ^a^	17.31 ± 0.02 ^c^	17.57 ± 0.15 ^bc^	17.21 ± 0.06 ^c^	17.62 ± 0.12 ^bc^	15.82 ± 0.01 ^d^	14.28 ± 0.17 ^e^	14.44 ± 0.03 ^e^
Cy	2.66 ± 0.09 ^bc^	2.56 ± 0.03 ^cd^	2.49 ± 0.00 ^cde^	2.96 ± 0.03 ^b^	2.68 ± 0.17 ^bc^	2.31 ± 0.02 ^def^	3.47 ± 0.05 ^a^	2.25 ± 0.12 ^ef^	2.13 ± 0.08 ^f^	2.23 ± 0.04 ^ef^
Pt	17.64 ± 0.39 ^a^	17.65 ± 0.16 ^a^	17.09 ± 0.02 ^abc^	17.35 ± 0.06 ^ab^	16.68 ± 0.15 ^bc^	17.20 ± 0.12 ^abc^	16.47 ± 0.17 ^c^	14.35 ± 0.29 ^d^	13.64 ± 0.17 ^d^	13.85 ± 0.04 ^d^
Pn	14.33 ± 0.34 ^a^	13.39 ± 0.31 ^b^	13.42 ± 0.11 ^b^	12.15 ± 0.10 ^c^	13.34 ± 0.37 ^b^	12.40 ± 0.02 ^c^	13.70 ± 0.31 ^ab^	12.29 ± 0.10 ^c^	11.90 ± 0.04 ^c^	12.37 ± 0.02 ^c^
Mv	92.36 ± 0.77 ^a^	93.87 ± 0.38 ^a^	85.15 ± 0.51 ^b^	85.93 ± 0.13 ^b^	85.88 ± 0.60 ^b^	85.71 ± 1.01 ^b^	84.15 ± 0.14 ^bc^	82.20 ± 0.56 ^c^	82.27 ± 0.12 ^c^	82.19 ± 0.18 ^c^
PnAc	4.76 ± 0.16 ^a^	2.68 ± 0.22 ^c^	4.55 ± 0.07 ^a^	2.83 ± 0.07 ^c^	4.42 ± 0.14 ^a^	2.73 ± 0.13 ^c^	4.66 ± 0.31 ^a^	3.73 ± 0.12 ^b^	3.26 ± 0.03 ^b^	3.08 ± 0.06 ^c^
MvAc	25.35 ± 0.74 ^a^	24.73 ± 0.15 ^ab^	23.75 ± 0.10 ^bc^	22.67 ± 0.08 ^c^	23.50 ± 0.74 ^bc^	23.58 ± 0.31 ^bc^	22.31 ± 0.08 ^c^	22.34 ± 0.33 ^c^	18.44 ± 0.21 ^c^	15.11 ± 0.07 ^d^
PnCm	2.26 ± 0.06 ^a^	2.27 ± 0.15 ^a^	2.18 ± 0.01 ^a^	2.27 ± 0.00 ^a^	2.09 ± 0.09 ^ab^	2.29 ± 0.01 ^a^	2.27 ± 0.14 ^a^	2.15 ± 0.02 ^a^	1.49 ± 0.05 ^c^	1.85 ± 0.03 ^b^
MvCm	8.81 ± 0.28 ^ab^	8.96 ± 0.16 ^a^	8.46 ± 0.05 ^abc^	7.96 ± 0.05 ^cd^	8.12 ± 0.25 ^cd^	7.78 ± 0.30 ^d^	8.86 ± 0.01 ^ab^	8.23 ± 0.13 ^bcd^	6.74 ± 0.04 ^e^	6.74 ± 0.13 ^e^
∑ FA	186.6 ± 1.6 ^a^	184.0 ± 0.1 ^a^	175.3 ± 1.0 ^b^	171.4 ± 0.4 ^c^	174.3 ± 1.1 ^bc^	171.2 ± 0.6 ^c^	173.5 ± 0.5 ^bc^	163.4 ± 1.0 ^d^	154.2 ± 0.3 ^e^	151.9 ± 0.1 ^e^
Fl (mg/L)										
Pro B1	33.65 ± 0.14 ^a^	33.78 ± 0.56 ^a^	33.35 ± 0.01 ^a^	32.02 ± 0.08 ^ab^	32.96 ± 0.22 ^a^	32.60 ± 0.62 ^ab^	31.98 ± 1.11 ^ab^	32.74 ± 0.75 ^ab^	32.06 ± 0.52 ^ab^	30.77 ± 0.47 ^b^
Cat	52.89 ± 0.55 ^a^	52.57 ± 0.30 ^a^	51.47 ± 0.73 ^ab^	51.16 ± 0.05 ^ab^	51.52 ± 0.43 ^ab^	51.02 ± 0.65 ^ab^	50.45 ± 0.14 ^ab^	50.81 ± 1.75 ^ab^	50.34 ± 0.59 ^ab^	49.51 ± 0.92 ^b^
Pro B2	35.84 ± 0.39 ^a^	35.78 ± 0.51 ^a^	35.59 ± 2.49 ^a^	33.60 ± 1.37 ^ab^	35.30 ± 2.09 ^a^	33.79 ± 2.23 ^ab^	32.47 ± 1.23 ^ab^	32.27 ± 0.92 ^ab^	28.95 ± 0.04 ^b^	28.91 ± 0.55 ^b^
Epicat	51.43 ± 1.30 ^a^	47.98 ± 2.14 ^ab^	46.17 ± 2.30 ^b^	45.65 ± 1.18 ^b^	46.04 ± 0.62 ^b^	45.90 ± 0.53 ^b^	43.59 ± 0.12 ^b^	45.79 ± 0.29 ^b^	45.69 ± 0.97 ^b^	43.46 ± 1.12 ^b^
Pro B3	4.41 ± 0.18 ^a^	4.37 ± 0.06 ^a^	4.28 ± 0.00 ^ab^	4.18 ± 0.15 ^ab^	4.22 ± 0.07 ^ab^	4.11 ± 0.06 ^ab^	4.07 ± 0.08 ^ab^	4.07 ± 0.02 ^ab^	4.06 ± 0.05 ^ab^	3.95 ± 0.02 ^b^
Pro B4	10.30 ± 0.49 ^a^	10.06 ± 0.07 ^a^	9.85 ± 0.51 ^a^	9.49 ± 0.42 ^ab^	9.30 ± 0.22 ^ab^	8.50 ± 0.26 ^ab^	7.62 ± 0.56 ^ab^	8.45 ± 0.83 ^ab^	7.63 ± 1.78 ^ab^	7.02 ± 0.34 ^b^
Pro C1	12.47 ± 0.31 ^a^	11.55 ± 0.85 ^ab^	10.51 ± 0.49 ^bc^	10.03 ± 0.06 ^bc^	10.47 ± 0.03 ^bc^	9.81 ± 0.18 ^bc^	9.70 ± 0.75 ^bc^	9.62 ± 0.56 ^c^	9.46 ± 0.46 ^c^	8.94 ± 0.31 ^c^
∑ Fl	201.0 ± 1.1 ^a^	196.1 ± 0.5 ^ab^	191.2 ± 6.5 ^abc^	186.1 ± 0.2 ^bcd^	189.8 ± 2.3 ^bc^	185.7 ± 4.0 ^bcd^	179.9 ± 0.4 ^cde^	183.8 ± 3.4 ^cd^	178.2 ± 2.3 ^de^	172.6 ± 1.0 ^e^
Color										
L^*^	14.6 ± 0.2 ^e^	14.6 ± 0.1 ^de^	15.0 ± 0.1 ^bc^	14.8 ± 0.1 ^cde^	15.0 ± 0.0 ^bc^	15.1 ± 0.1 ^bc^	15.2 ± 0.0 ^b^	16.4 ± 0.1 ^a^	16.5 ± 0.2 ^a^	16.3 ± 0.1 ^a^
a^*^	45.8 ± 0.1 ^e^	46.0 ± 0.1 ^cde^	46.2 ± 0.1 ^cde^	45.9 ± 0.1 ^de^	46.3 ± 0.0 ^bcd^	46.4 ± 0.1 ^bc^	46.1 ± 0.3 ^b^	47.9 ± 0.0 ^a^	47.9 ± 0.2 ^a^	47.7 ± 0.2 ^a^
b^*^	24.8 ± 0.1 ^d^	25.4 ± 0.2 ^bc^	25.3 ± 0.1 ^bcd^	25.0 ± 0.1 ^cd^	25.5 ± 0.0 ^bc^	25.6 ± 0.1 ^b^	25.3 ± 0.1 ^bcd^	27.7 ± 0.1 ^a^	27.7 ± 0.2 ^a^	27.4 ± 0.2 ^a^
C^*^	52.2 ± 0.1 ^e^	52.4 ± 0.2 ^cde^	52.7 ± 0.1 ^cde^	52.3 ± 0.1 ^de^	52.8 ± 0.0 ^bcd^	53.0 ± 0.1 ^bc^	53.4 ± 0.0 ^b^	55.3 ± 0.1 ^a^	55.3 ± 0.3 ^a^	55.0 ± 0.3 ^a^
H^*^	0.5 ± 0.0 ^d^	0.5 ± 0.0 ^cd^	0.5 ± 0.0 ^cd^	0.5 ± 0.0 ^bc^	0.5 ± 0.0 ^cd^	0.5 ± 0.0 ^cd^	0.5 ± 0.0 ^bc^	0.5 ± 0.0 ^a^	0.5 ± 0.00 ^a^	0.5 ± 0.0 ^ab^
∆E^*^	-	0.6	0.7	0.4	1.0	1.2	0.9	4.0	4.1	3.7

^a–g^ Different letters in the same row show significant difference (*p* < 0.05) among the samples. RW: red wine; TP: total phenolics; TA: total anthocyanins; TT: total tannins; FA: free anthocyanins (Dph: delphinidin-3-*O*-glucoside; Cy: cyanidin-3-*O*-glucoside; Pt: petunidin-3-*O*-glucoside; Pn: peonidin-3-*O*-glucoside; Mv: malvidin-3-*O*-glucoside; PnAc: peonidin-3-*O*-glucoside acetate; MvAc: malvidin-3-*O*-glucoside acetate; PnCm: peonidin-3-*O*-glucoside *p*-coumarate; MvCm: malvidin-3-*O*-glucoside *p*-coumarate); Fl: flavanols; Pro: procyanidin; Cat: (+)-catechin; Epicat: (−)-epicatechin.

**Table 2:** Phenolic profile and color parameters of pressurized and unpressurized white wine samples.

Analysis	WW	High Hydrostatic Pressure Processing								
	Untreated	200 MPa/5 min	200 MPa/15 min	200 MPa/25 min	400 MPa/5 min	400 MPa/15 min	400 MPa/25 min	600 MPa/5 min	600 MPa/15 min	600 MPa/25 min
TP (mg/L)	261.7 ± 0.3 ^a^	259.1 ± 0.5 ^abc^	258.7 ± 1.1 ^abcd^	256.5 ± 0.5 ^cd^	256.1 ± 0.3 ^d^	258.8 ± 0.8 ^abcd^	256.1 ± 0.5 ^cd^	259.7 ± 0.1 ^ab^	257.1 ± 1.1 ^bcd^	256.6 ± 1.4 ^cd^
Pa (mg/L)										
Gal	2.56 ± 0.01 ^de^	2.66 ± 0.02 ^a^	2.63 ± 0.00 ^ab^	2.62 ± 0.02 ^abc^	2.64 ± 0.01 ^a^	2.61 ± 0.01 ^abcd^	2.57 ± 0.01 ^cde^	2.58 ± 0.02 ^bcde^	2.56 ± 0.01 ^de^	2.55 ± 0.01 ^e^
Protocat	5.67 ± 0.02 ^bc^	5.91 ± 0.11 ^a^	5.89 ± 0.02 ^a^	5.77 ± 0.03 ^ab^	5.75 ± 0.09 ^ab^	5.72 ± 0.01 ^ab^	5.64 ± 0.02 ^bc^	5.63 ± 0.03 ^bc^	5.61 ± 0.00 ^bc^	5.48 ± 0.00 ^c^
Van	0.78 ± 0.06 ^a^	0.72 ± 0.10 ^a^	0.52 ± 0.04 ^bc^	0.51 ± 0.05 ^c^	0.70 ± 0.06 ^ab^	0.51 ± 0.01 ^bc^	0.49 ± 0.00 ^c^	0.41 ± 0.03 ^c^	0.40 ± 0.00 ^c^	0.38 ± 0.00 ^c^
Syr	0.25 ± 0.02 ^a^	0.25 ± 0.03 ^a^	0.22 ± 0.03 ^ab^	0.19 ± 0.00 ^ab^	0.23 ± 0.01 ^ab^	0.20 ± 0.00 ^ab^	0.18 ± 0.03 ^ab^	0.19 ± 0.00 ^ab^	0.18 ± 0.01 ^ab^	0.17 ± 0.01 ^b^
Caft	30.61 ± 0.25 ^a^	30.47 ± 0.02 ^a^	29.69 ± 0.26 ^bcd^	29.25 ± 0.01 ^ef^	30.38 ± 0.02 ^ab^	29.47 ± 0.12 ^cde^	28.88 ± 0.03 ^ef^	30.21 ± 0.01 ^abc^	28.76 ± 0.40 ^ef^	28.59 ± 0.25 ^f^
Chlo	2.40 ± 0.00 ^b^	2.44 ± 0.00 ^a^	2.39 ± 0.02 ^b^	2.37 ± 0.01 ^b^	2.39 ± 0.02 ^b^	2.36 ± 0.00 ^b^	2.30 ± 0.00 ^c^	2.37 ± 0.00 ^b^	2.28 ± 0.02 ^c^	2.26 ± 0.01 ^c^
Caf	2.30 ± 0.01 ^b^	2.41 ± 0.01 ^a^	2.27 ± 0.01 ^bc^	2.26 ± 0.01 ^bc^	2.28 ± 0.01 ^bc^	2.25 ± 0.02 ^bc^	2.24 ± 0.02 ^cd^	2.24 ± 0.00 ^cd^	2.20 ± 0.02 ^d^	2.20 ± 0.00 ^d^
p-Coum	1.43 ± 0.01 ^bc^	1.49 ± 0.01 ^a^	1.47 ± 0.01 ^ab^	1.45 ± 0.01 ^abc^	1.49 ± 0.01 ^a^	1.46 ± 0.01 ^abc^	1.43 ± 0.01 ^bc^	1.45 ± 0.02 ^abc^	1.44 ± 0.01 ^bc^	1.42 ± 0.02 ^c^
Fer	0.57 ± 0.01 ^a^	0.58 ± 0.03 ^a^	0.56 ± 0.00 ^a^	0.56 ± 0.00 ^a^	0.56 ± 0.00 ^a^	0.56 ± 0.01 ^a^	0.55 ± 0.00 ^a^	0.56 ± 0.01 ^a^	0.56 ± 0.00 ^a^	0.55 ± 0.01 ^a^
∑ Pa	46.6 ± 0.3 ^a^	46.9 ± 0.1 ^a^	45.6 ± 0.3 ^b^	45.0 ± 0.1 ^bc^	46.4 ± 0.1 ^a^	45.1 ± 0.2 ^b^	44.3 ± 0.0 ^cd^	45.7 ± 0.1 ^b^	44.0 ± 0.3 ^d^	43.6 ± 0.2 ^d^
Fl (mg/L)										
Pro B1	11.47 ± 0.01 ^a^	11.30 ± 0.00 ^ab^	11.18 ± 0.01 ^abc^	11.14 ± 0.06 ^abc^	11.29 ± 0.01 ^ab^	11.03 ± 0.01 ^bc^	10.84 ± 0.19 ^cd^	10.81 ± 0.10 ^cd^	10.64 ± 0.17 ^d^	10.55 ± 0.13 ^d^
ProB2	2.66 ± 0.12 ^a^	2.57 ± 0.16 ^a^	2.28 ± 0.09 ^ab^	2.00 ± 0.15 ^bc^	1.83 ± 0.02 ^c^	1.76 ± 0.14 ^c^	1.67 ± 0.09 ^c^	1.77 ± 0.09 ^c^	1.67 ± 0.08 ^c^	1.58 ± 0.02 ^c^
Cat	6.83 ± 0.04 ^a^	6.02 ± 0.21 ^b^	4.41 ± 0.22 ^c^	3.40 ± 0.05 ^de^	3.70 ± 0.05 ^d^	3.21 ± 0.05 ^ef^	3.12 ± 0.06 ^ef^	2.97 ± 0.02 ^f^	2.95 ± 0.06 ^f^	2.92 ± 0.04 ^f^
Epicat	10.53 ± 0.11 ^a^	10.24 ± 0.03 ^ab^	9.89 ± 0.14 ^bc^	9.50 ± 0.01 ^c^	9.67 ± 0.05 ^c^	8.88 ± 0.01 ^d^	8.77 ± 0.22 ^d^	8.68 ± 0.29 ^d^	8.55 ± 0.03 ^d^	7.83 ± 0.03 ^e^
∑ Fl	31.5 ± 0.0 ^a^	30.1 ± 0.4 ^b^	27.8 ± 0.0 ^c^	26.1 ± 0.1 ^d^	26.5 ± 0.1 ^d^	24.9 ± 0.2 ^e^	24.4 ± 0.1 ^ef^	24.2 ± 0.1 ^ef^	23.8 ± 0.1 ^f^	22.9 ± 0.2 ^g^
Color										
L^*^	101.8 ± 0.0 ^a^	101.2 ± 0.6 ^a^	101.1 ± 0.0 ^a^	100.1 ± 0.0 ^b^	100.0 ± 0.0 ^b^	99.9 ± 0.1 ^b^	100.0 ± 0.0 ^b^	98.2 ± 0.1 ^c^	98.1 ± 0.0 ^c^	98.0 ± 0.0 ^c^
a^*^	−0.2 ± 0.0 ^c^	−0.2 ± 0.1 ^bc^	−0.1 ± 0.0 ^bc^	−0.0 ± 0.0 ^ab^	0.0 ± 0.0 ^a^	0.0 ± 0.0 ^a^	0.0 ± 0.0 ^a^	−0.6 ± 0.1 ^d^	−0.6 ± 0.0 ^d^	−0.5 ± 0.1 ^d^
b^*^	−0.8 ± 0.0 ^c^	−0.5 ± 0.3 ^bc^	−0.5 ± 0.0 ^b^	−0.0 ± 0.0 ^a^	0.0 ± 0.0 ^a^	0.1 ± 0.1 ^a^	−0.0 ± 0.0 ^a^	−0.6 ± 0.0 ^bc^	−0.6 ± 0.0 ^bc^	−0.6 ± 0.0 ^bc^
C^*^	0.9 ± 0.0 ^a^	0.5 ± 0.3 ^ab^	0.5 ± 0.0 ^bc^	0.0 ± 0.0 ^d^	0.0 ± 0.0 ^d^	0.1 ± 0.0 ^cd^	0.1 ± 0.0 ^d^	0.6 ± 0.0 ^ab^	0.6 ± 0.0 ^ab^	0.6 ± 0.0 ^ab^
H^*^	1.3 ± 0.0 ^a^	1.3 ± 0.0 ^a^	1.3 ± 0.0 ^a^	1.0 ± 0.2 ^a^	0.1 ± 0.7 ^ab^	1.0 ± 0.4 ^a^	−0.8 ± 0.9 ^ab^	−1.5 ± 0.0 ^b^	−1.5 ± 0.0 ^b^	−1.5 ± 0.0 ^b^
∆E^*^	-	0.7	0.9	1.9	2.0	2.2	2.0	3.7	3.7	3.9

^a–g^ Different letters in the same row show significant difference (*p* < 0.05) among the samples. WW: white wine; TP: total phenolics; Pa: phenolic acids; Gal: gallic acid; Protocat: protocatechuic acid; Van: vanillic acid; Syr: syringic acid; Caft: caftaric acid; Chlo: chlorogenic acid; Caf: caffeic acid; *p*-Coum: *p*-coumaric acid; Fer: ferulic acid; Fl: flavanols; Pro: procyanidin; Cat: (+)-catechin; Epicat: (−)-epicatechin.

**Table 3:** The average scores for sensory attributes (color, odor and taste) of pressurized red and white wines.

**Time (months)**	**Red Wine**	**Color**	**Odor**	**Taste**
0	standard SO_2_	8.7 ± 0.5 ^a^	8.6 ± 0.5 ^a^	8.5 ± 0.5 ^a^
	low SO_2_+GSH	8.6 ± 0.5 ^a^	8.5 ± 0.5 ^a^	8.4 ± 0.5 ^a^
	low SO_2_	8.3 ± 0.5 ^a^	8.3 ± 0.5 ^a^	8.4 ± 0.5 ^a^
3	standard SO_2_	8.1 ± 0.3 ^a^	8.0 ± 0.5 ^a^	7.9 ± 0.3 ^a^
	low SO_2_+GSH	7.8 ± 0.3 ^ab^	7.7 ± 0.5 ^ab^	7.5 ± 0.3 ^a^
	low SO_2_	7.4 ± 0.5 ^b^	7.4 ± 0.3 ^b^	7.2 ± 0.5 ^a^
6	standard SO_2_	7.8 ± 0.5 ^a^	7.7 ± 0.5 ^a^	7.6 ± 0.5 ^a^
	low SO_2_+GSH	7.6 ± 0.4 ^ab^	7.4 ± 0.3 ^ab^	7.3 ± 0.3 ^ab^
	low SO_2_	7.2 ± 0.3 ^b^	7.0 ± 0.3 ^b^	6.9 ± 0.3 ^b^
12	standard SO_2_	7.4 ± 0.5 ^a^	7.2 ± 0.4 ^a^	6.8 ± 0.3 ^a^
	low SO_2_+GSH	7.1 ± 0.3 ^a^	6.9 ± 0.3 ^ab^	6.6 ± 0.4 ^ab^
	low SO_2_	6.6 ± 0.5 ^b^	6.5 ± 0.5 ^b^	6.1 ± 0.4 ^b^
**Time (months)**	**White Wine**	**Color**	**Odor**	**Taste**
0	standard SO_2_	8.5 ± 0.5 ^a^	8.3 ± 0.5 ^a^	8.2 ± 0.3 ^a^
	low SO_2_+GSH	8.0 ± 0.4 ^b^	7.8 ± 0.4 ^ab^	7.7 ± 0.4 ^a^
	low SO_2_	7.9 ± 0.3 ^b^	7.7 ± 0.5 ^b^	7.6 ± 0.5 ^a^
3	standard SO_2_	7.5 ± 0.5 ^a^	7.3 ± 0.5 ^a^	7.2 ± 0.4 ^a^
	low SO_2_+GSH	6.9 ± 0.3 ^b^	6.7 ± 0.5 ^b^	6.6 ± 0.5 ^b^
	low SO_2_	6.8 ± 0.4 ^b^	6.6 ± 0.3 ^b^	6.3 ± 0.5 ^b^
6	standard SO_2_	7.3 ± 0.3 ^a^	7.2 ± 0.4 ^a^	7.0 ± 0.3 ^a^
	low SO_2_+GSH	6.7 ± 0.4 ^b^	6.6 ± 0.5 ^b^	6.5 ± 0.5 ^ab^
	low SO_2_	6.6 ± 0.5 ^b^	6.5 ± 0.5 ^b^	6.2 ± 0.4 ^b^
12	standard SO_2_	6.9 ± 0.4 ^a^	6.6 ± 0.3 ^a^	6.5 ± 0.5 ^a^
	low SO_2_+GSH	6.4 ± 0.5 ^ab^	6.0 ± 0.4 ^ab^	5.8 ± 0.4 ^b^
	low SO_2_	6.1 ± 0.3 ^b^	5.8 ± 0.3 ^b^	5.8 ± 0.4 ^b^

^a^^,^^b^ The samples with different letters are statistically significant (*p* < 0.05).

## References

[1] Van Wyk S., Silva F.V. (2019). Nonthermal Preservation of Wine. Preservatives and Preservation Approaches in Beverages.

[2] Muntean M.-V., Marian O., Barbieru V., Cătunescu G.M., Ranta O., Drocas I., Terhes S. (2016). High pressure processing in food industry–characteristics and applications. Agric. Agric. Sci. Procedia.

[3] Puig A., Vilavella M., Daoudi L., Guamis B., Minguez S. (2003). Microbiological and biochemical stabilization of wines by application of the high pressure technique. Bull. OIV Fr..

[4] Buzrul S. (2012). High hydrostatic pressure treatment of beer and wine: A review. Innov. Food Sci. Emerg. Technol..

[5] González-Arenzana L., Sevenich R., Rauh C., López R., Knorr D., López-Alfaro I. (2016). Inactivation of Brettanomyces bruxellensis by high hydrostatic pressure technology. Food Control.

[6] Van Wyk S., Silva F.V. (2017). High pressure processing inactivation of *Brettanomyces bruxellensis* in seven different table wines. Food Control.

[7] Van Wyk S., Silva F.V. (2017). High pressure inactivation of *Brettanomyces bruxellensis* in red wine. Food Microbiol..

[8] Mok C., Song K., Park Y., Lim S., Ruan R., Chen P. (2006). High hydrostatic pressure pasteurization of red wine. J. Food Sci..

[9] Santos M.C., Nunes C., Jourdes M., Teissedre P.-L., Rodrigues A., Amado O., Saraiva J.A., Coimbra M.A. (2016). Evaluation of the potential of high pressure technology as an enological practice for red wines. Innov. Food Sci. Emerg. Technol..

[10] Tao Y., Sun D.-W., Górecki A., Błaszczak W., Lamparski G., Amarowicz R., Fornal J., Jeliński T. (2012). Effects of high hydrostatic pressure processing on the physicochemical and sensorial properties of a red wine. Innov. Food Sci. Emerg. Technol..

[11] Santos M.C., Nunes C., Cappelle J., Gonçalves F.J., Rodrigues A., Saraiva J.A., Coimbra M.A. (2013). Effect of high pressure treatments on the physicochemical properties of a sulphur dioxide-free red wine. Food Chem..

[12] Santos M.C., Nunes C., Rocha M.A.M., Rodrigues A., Rocha S.M., Saraiva J.A., Coimbra M.A. (2013). Impact of high pressure treatments on the physicochemical properties of a sulphur dioxide-free white wine during bottle storage: Evidence for Maillard reaction acceleration. Innov. Food Sci. Emerg. Technol..

[13] Santos M.C., Nunes C., Rocha M.A.M., Rodrigues A., Rocha S.M., Saraiva J.A., Coimbra M.A. (2015). High pressure treatments accelerate changes in volatile composition of sulphur dioxide-free wine during bottle storage. Food Chem..

[14] Sun X., Li L., Ma T., Zhao F., Yu D., Huang W., Zhan J. (2016). High hydrostatic pressure treatment: An artificial accelerating aging method which did not change the region and variety non-colored phenolic characteristic of red wine. Innov. Food Sci. Emerg. Technol..

[15] Tao Y., Sun D.-W., Górecki A., Błaszczak W., Lamparski G., Amarowicz R., Fornal J., Jeliński T. (2016). A preliminary study about the influence of high hydrostatic pressure processing in parallel with oak chip maceration on the physicochemical and sensory properties of a young red wine. Food Chem..

[16] Norton T., Sun D.-W. (2008). Recent advances in the use of high pressure as an effective processing technique in the food industry. Food Bioprocess Technol..

[17] Ferrer-Gallego R., Puxeu M., Martín L., Nart E., Hidalgo C., Andorrà I. (2017). Microbiological, Physical, and Chemical Procedures to Elaborate High-Quality SO_2_-Free Wines. Grapes and Wines-Advances in Production, Processing, Analysis and Valorization.

[18] Vally H., Misso N.L., Madan V. (2009). Clinical effects of sulphite additives. Clin. Exp. Allergy.

[19] Briones-Labarca V., Perez-Wom M., Habib G., Giovagnoli-Vicuña C., Cañas-Sarazua R., Tabilo-Munizaga G., Salazar F.N. (2017). Oenological and quality characteristic on young white wines (sauvignon blanc): Effects of high hydrostatic pressure processing. J. Food Qual..

[20] Van Wyk S., Farid M.M., Silva F.V. (2018). SO2, high pressure processing and pulsed electric field treatments of red wine: Effect on sensory, Brettanomyces inactivation and other quality parameters during one year storage. Innov. Food Sci. Emerg. Technol..

[21] Christofi S., Malliaris D., Katsaros G., Panagou E., Kallithraka S. (2020). Limit SO_2_ content of wines by applying High Hydrostatic Pressure. Innov. Food Sci. Emerg. Technol..

[22] Guerrero R.F., Cantos-Villar E. (2015). Demonstrating the efficiency of sulphur dioxide replacements in wine: A parameter review. Trends Food Sci. Technol..

[23] Lisanti M.T., Blaiotta G., Nioi C., Moio L. (2019). Alternative Methods to SO_2_ for Microbiological Stabilization of Wine. Compr. Rev. Food Sci. Food Saf..

[24] Kritzinger E.C., Bauer F.F., Du Toit W.J. (2012). Role of glutathione in winemaking: A review. J. Agric. Food Chem..

[25] El Hosry L., Auezova L., Sakr A., Hajj-Moussa E. (2009). Browning susceptibility of white wine and antioxidant effect of glutathione. Int. J. Food Sci. Technol..

[26] Oliveira C.M., Ferreira A.C.S., De Freitas V., Silva A.M. (2011). Oxidation mechanisms occurring in wines. Food Res. Int..

[27] Nikolantonaki M., Julien P., Coelho C., Roullier-Gall C., Ballester J., Schmitt-Kopplin P., Gougeon R.D. (2018). Impact of glutathione on wines oxidative stability: A combined sensory and metabolomic study. Front. Chem..

[28] OIV (2009). Compendium of International Methods of Wine and Must Analysis.

[29] Lukić K., Tomašević M., Ćurko N., Sivrić A., Ružman E., Kovačević Ganić K. (2019). Influence of non-thermal processing techniques on sulfur dioxide and oxygen concentrations in young and aged wines. Croat. J. Food Technol. Biotechnol. Nutr..

[30] Nunes C., Santos M.C., Saraiva J.A., Rocha S.M., Coimbra M.A. (2017). Influence of high hydrostatic pressure technology on wine chemical and sensorial characteristics: Potentialities and drawbacks. Advances in Food and Nutrition Research.

[31] Singleton V.L., Rossi J.A. (1965). Colorimetry of total phenolics with phosphomolybdic-phosphotungstic acid reagents. Am. J. Enol. Vitic..

[32] Ribéreau-Gayon P., Stonestreet E. (1965). Determination of anthocyanins in red wine. Bull. Soc. Chim. Fr..

[33] Ribéreau-Gayon P., Stonestreet E. (1966). Concentration of the tannins in red wine and determination of their structure. Chim. Anal..

[34] Lorrain B., Chira K., Teissedre P.-L. (2011). Phenolic composition of Merlot and Cabernet-Sauvignon grapes from Bordeaux vineyard for the 2009-vintage: Comparison to 2006, 2007 and 2008 vintages. Food Chem..

[35] Lukić K., Brnčić M., Ćurko N., Tomašević M., Valinger D., Denoya G.I., Barba F.J., Kovačević Ganić K. (2019). Effects of high power ultrasound treatments on the phenolic, chromatic and aroma composition of young and aged red wine. Ultrason. Sonochem..

[36] Ćurko N., Kovačević Ganić K., Gracin L., Đapić M., Jourdes M., Teissedre P.-L. (2014). Characterization of seed and skin polyphenolic extracts of two red grape cultivars grown in Croatia and their sensory perception in a wine model medium. Food Chem..

[37] Monagas M., Suárez R., Gómez-Cordovés C., Bartolomé B. (2005). Simultaneous determination of nonanthocyanin phenolic compounds in red wines by HPLC-DAD/ESI-MS. Am. J. Enol. Vitic..

[38] Lukić K., Brnčić M., Ćurko N., Tomašević M., Tušek A.J., Kovačević Ganić K. (2020). Quality characteristics of white wine: The short-and long-term impact of high power ultrasound processing. Ultrason. Sonochem..

[39] Tomašević M., Gracin L., Ćurko N., Kovačević Ganić K. (2017). Impact of pre-fermentative maceration and yeast strain along with glutathione and SO_2_ additions on the aroma of *Vitis vinifera* L. Pošip wine and its evaluation during bottle aging. LWT.

[40] Stone H., Sidel J.L. (1993). Sensory Evaluation Practices.

[41] Tao Y., Wu D., Sun D.-W., Górecki A., Błaszczak W., Fornal J., Jeliński T. (2013). Quantitative and predictive study of the evolution of wine quality parameters during high hydrostatic pressure processing. Innov. Food Sci. Emerg. Technol..

[42] Chen X., Li L., You Y., Mao B., Zhao W., Zhan J. (2012). The effects of ultra-high pressure treatment on the phenolic composition of red wine. S. Afr. J. Enol. Vitic..

[43] Sun X., Chen X., Li L., Ma T., Zhao F., Huang W., Zhan J. (2015). Effect of ultra-high pressure treatment on the chemical properties, colour and sensory quality of young red wine. S. Afr. J. Enol. Vitic..

[44] Martínez J., Melgosa M., Pérez M., Hita E., Negueruela A. (2001). Note. Visual and instrumental color evaluation in red wines. Food Sci. Technol. Int..

[45] Tomašević M., Lukić K., Bosiljkov T., Kelšin K., Ćurko N., Kovačević Ganić K. (2017). Effect of high hydrostatic pressure on the volatile compounds in wine. Rad. Poljopr. Fak. Univ. U Sarajev. Fac. Agric. Univ. Sarajevo.

[46] Monagas M., Bartolomé B., Gómez-Cordovés C. (2005). Evolution of polyphenols in red wines from *Vitis vinifera* L. during aging in the bottle. Eur. Food Res. Technol..

[47] Monagas M., Gómez-Cordovés C., Bartolomé B. (2006). Evolution of the phenolic content of red wines from *Vitis vinifera* L. during ageing in bottle. Food Chem..

[48] Santos M.C., Nunes C., Ferreira A.S., Jourdes M., Teissedre P.-L., Rodrigues A., Amado O., Saraiva J.A., Coimbra M.A. (2019). Comparison of high pressure treatment with conventional red wine aging processes: Impact on phenolic composition. Food Res. Int..

[49] Gallego M.G., García-Carpintero E.G., Sánchez-Palomo E., Viñas M.G., Hermosín-Gutiérrez I. (2013). Evolution of the phenolic content, chromatic characteristics and sensory properties during bottle storage of red single-cultivar wines from Castilla La Mancha region. Food Res. Int..

[50] Ribéreau-Gayon P., Glories Y., Maujean A., Dubourdieu D. (2006). Handbook of Enology, Volume 2: The Chemistry of Wine-Stabilization and Treatments.

[51] Coetzee C., Du Toit W. (2015). Sauvignon blanc wine: Contribution of ageing and oxygen on aromatic and non-aromatic compounds and sensory composition-A review. S. Afr. J. Enol. Vitic..

[52] Pérez-Prieto L., López-Roca J., Gómez-Plaza E. (2003). Differences in major volatile compounds of red wines according to storage length and storage conditions. J. Food Compos. Anal..

[53] Garde-Cerdán T., Ancín-Azpilicueta C. (2007). Effect of SO_2_ on the formation and evolution of volatile compounds in wines. Food Control.

[54] Nikolantonaki M., Waterhouse A.L. (2012). A method to quantify quinone reaction rates with wine relevant nucleophiles: A key to the understanding of oxidative loss of varietal thiols. J. Agric. Food Chem..

[55] Kallithraka S., Salacha M., Tzourou I. (2009). Changes in phenolic composition and antioxidant activity of white wine during bottle storage: Accelerated browning test versus bottle storage. Food Chem..

[56] Hernanz D., Gallo V., Recamales Á.F., Meléndez-Martínez A.J., González-Miret M.L., Heredia F.J. (2009). Effect of storage on the phenolic content, volatile composition and colour of white wines from the varieties Zalema and Colombard. Food Chem..

[57] Ferreira-Lima N.E., Burin V.M., Caliari V., Bordignon-Luiz M.T. (2016). Impact of pressing conditions on the phenolic composition, radical scavenging activity and glutathione content of Brazilian *Vitis vinifera* white wines and evolution during bottle ageing. Food Bioprocess Technol..

[58] Di Lecce G., Boselli E., D’Ignazi G., Frega N.G. (2013). Evolution of phenolics and glutathione in Verdicchio wine obtained with maceration under reductive conditions. LWT.

[59] Recamales Á.F., Sayago A., González-Miret M.L., Hernanz D. (2006). The effect of time and storage conditions on the phenolic composition and colour of white wine. Food Res. Int..

[60] Sonni F., Clark A.C., Prenzler P.D., Riponi C., Scollary G.R. (2011). Antioxidant action of glutathione and the ascorbic acid/glutathione pair in a model white wine. J. Agric. Food Chem..

[61] Antoce A.O., Badea G.A., Cojocaru G.A. (2016). Effects of glutathione and ascorbic acid addition on the CIELab chromatic characteristics of Muscat Ottonel wines. Agric. Agric. Sci. Procedia.

[62] Roussis I.G., Sergianitis S. (2008). Protection of some aroma volatiles in a model wine medium by sulphur dioxide and mixtures of glutathione with caffeic acid or gallic acid. Flavour Fragr. J..

[63] Kilmartin P.A. (2009). The oxidation of red and white wines and its impact on wine aroma. Chem. N. Z..

[64] Panero L., Motta S., Petrozziello M., Guaita M., Bosso A. (2015). Effect of SO 2, reduced glutathione and ellagitannins on the shelf life of bottled white wines. Eur. Food Res. Technol..

[65] Waterhouse A.L., Frost S., Ugliano M., Cantu A.R., Currie B.L., Anderson M., Chassy A.W., Vidal S., Diéval J.-B., Aagaard O. (2016). Sulfur dioxide–oxygen consumption ratio reveals differences in bottled wine oxidation. Am. J. Enol. Vitic..

[66] Roussis I., Patrianakou M., Drossiadis A. (2013). Protection of aroma volatiles in a red wine with low sulphur dioxide by a mixture of glutathione, caffeic acid and gallic acid. S. Afr. J. Enol. Vitic..

[67] Pati S., Crupi P., Savastano M.L., Benucci I., Esti M. (2020). Evolution of phenolic and volatile compounds during bottle storage of a white wine without added sulfite. J. Sci. Food Agric..

[68] Webber V., Dutra S.V., Spinelli F.R., Carnieli G.J., Cardozo A., Vanderlinde R. (2017). Effect of glutathione during bottle storage of sparkling wine. Food Chem..

[69] Fuhrman B., Volkova N., Suraski A., Aviram M. (2001). White wine with red wine-like properties: Increased extraction of grape skin polyphenols improves the antioxidant capacity of the derived white wine. J. Agric. Food Chem..

[70] Forde C.G., Cox A., Williams E.R., Boss P.K. (2011). Associations between the Sensory Attributes and Volatile Composition of Cabernet Sauvignon Wines and the Volatile Composition of the Grapes Used for Their Production. J. Agric. Food Chem..

